# Medical News and Miscellany

**Published:** 1889-09

**Authors:** 


### Medical News and Miscellany.

Dr. Justus Duffau has removed from Austin to Houston.

Dr. C. T. Copoer has removed from Lilac, Texas, to Boone, Iowa.

Dr. T. D. Wooten and family spent the summer at Virginia Springs. Lucky doctor.

Dr. T. J. Tyner and wife are making a northern tour through New York and Canada—to return in October.

Dr. M. L. Haggard has removed from Mountain Peak to Midlothian. Dr. H. B. Talliafero has removed to Bruceville.

Dr. Frank Rainey, Superintendent State Blind Institute, Austin, has recently been very sick, but we are pleased to say is all right again.

Removals.—Dr. B. H. Harris, late of Morris, Dallas county, has removed to Okla-Union, Wilbarger county; Dr. L. W. Cocke, from San Marcos to Boerne.

Married—In Eminence, Ky., September 11 inst., Dr. C. M. Rosser, of Dallas, Texas, to Miss Alma Curtice, of Louisville. We acknowledge the courtesy of a card.

"Can you support a family, young man?" asked *Pater Familias* of young Canesucker, a suitor of his daughter.

"*I only wanted* Sarah," he said dejectedly.

Dr. Merry-man asserts that Dr. Wile, of the New England Medical Monthly, never was a single man, and never can be, because he's always been *double* you see (W. C.)

DR. W. M. YANDELL has been appointed "Health Physician" of El Paso, as successor to himself as Health Officer, and to Dr. —— as City Physician—a consolidation. A good appointment.

DR. W. P. FLEMING, lately of Georgetown, Texas, and recently appointed chief surgeon of the Columbia & Puget Sound railway, has left Franklin, W. T., and settled in San Diego, Cal.

WE REGRET to learn that Dr. L. B. Creath, of Kinneyville, has scarlet fever of a malignant type among his children. The doctor has our sympathy and sincere wishes for the recovery of his dear little ones.

FOR SALE.—Several trios of fine young genuine Plymoth Rock fowls, spring chickens, of the famous Heyflebower prize strain. Five Dollars per trio, on board cars. Single cockerels $2.50. Address editor this journal.

DIED—In Terrell, Texas, September —, Dr. Jas. H. Webb, one of the oldest practicing physicians in the State. Dr. Webb was at one time, '67-'8, connected with the Texas Medical College, as Professor of Materia Medica.

SUBSCRIBERS to the JOURNAL, whose time has expired, are earnestly requested to promptly renew their subscriptions; and those in arrears are earnestly requested to pay up. It takes money, and much of it, to run a journal like this.

DIED—August 29, ult., William Werner, young son of Dr. and Mrs. L. B. Creath, of Kinneyville, Texas. This bright and promising little fellow died of scarlet fever, after days of suffering. We tender to the stricken parents our sincerest sympathy.

Dr. R. T. KNOX, of whose illness we made mention in our last number, has been compelled to give up work altogether, and has retired to Kerrville—a cool mountain resort on the Guadalupe

—to recuperate—and catch bass. Later.—The doctor has returned to Gonzales much improved.

---

THE C. O. P. Co., of 60 Broad street, New York, manufacture a "vegetable lard," from the oil of cotton seed, which is pure and sweet. See ad.

---

THE JOURNAL is officially informed of the expulsion of Dr. H. W. Brown and his son, Dr. R. E. Brown, from the Waco Medical Society, for gross violation of the code of ethics, in aligning themselves with a lot of advertising quacks, an act which the JOURNAL highly approves.

---

THE AUSTIN DISTRICT MEDICAL ASSOCIATION is in quarterly session in this city, with a large attendance, as we go to press. A full report will be secured for the JOURNAL.

---

BIOGRAPHY OF CONTEMPORARY PHYSICIANS OF TEXAS.—This work is meeting with much encouragement, and already the subscription list is distinctly representative of the Texas profession. The portraits of a large number of leading men have been secured, and many more promised. Subscribe now, ere it is too late.

---

DR. T. J. HEARD, of Galveston, the first President of the Texas State Medical Association, and perhaps the oldest living practitioner in Texas, he having begun practice in Washington county in '37, has written the opening chapter for "Biography of Contemporary Physicians of Texas," it being an account of the practice and of his colleagues away back in the forties, and long before the advent of railroads. etc. It leads up to the organization of the State Association, and is most interesting reading.

---

DR. WM. CASTON, late of Corsicana, Texas, it will be remembered, removed recently to Spokane Falls, W. T. He did so just in time to catch the fire, or be caught by the fire, which destroyed the city. The doctor writes us that he lost every vestige of outfit, consisting of a valuable library and medical and surgical armamentarium, and had no insurance. We extend to him our hearty sympathy, and his many friends in Texas and Mississippi will be sorry to hear of his bad luck. But the doctor is young and full of energy, and Spokane offers a splendid field, so he says, and no doubt he will soon be "on deck" and all right again. We hope so.

---

WASTED THUNDER.—And now comes the Medical Standard, of Chicago, and says the criticism at which we took umbrage—and which elicited the article "Bush-whacking," in our last issue, was not intended for us at all, but for a "local contemporary," to-wit, the North American Ppactioner, the "organ" of the Chicago Post-Graduate School. It seems that we and the Standard were shooting at the same target, and we thought we had gotten the charge instead of the Practitioner. This is just one of those situations which have to be made the most of. We don't know what to say, and suppose that the less said the better; but we have wasted some very choice thunder—all the same. We beg pardon of the Standard, and its "concealed fellow," "whoever he, she or it may be."

---

EDITORS OFF DUTY.—The wittiest thing said at the banquet and "clam-bake" tendered the Association of American Medical Editors by the "Squantam Club" at Danbury lately, was by a gentleman, who, in responding to a toast, and referring to the fact that all the speakers had eulogized the clam, said: "But we came to bury Cæsar, not to praise him."

Comparing the swallowing of a clam to the interment of the great Roman, and paraphrasing Marc Anthony's oration is highly complimentary to the clam, but Dr. Merryman says, it is the most un-Cæsar-nable joke he ever heard.

The *New England Medical Monthly* gives *in extenso* the toasts and responses upon that occasion. We think we never saw such and so many attempts at wit; they ranged all the way from "good" to "bad," including "indifferent" and "chestnut"; puns on "Love," "Wile" and "clam" largely predominating. "Brother (ly) Love" certainly was a conspicuous feature on that occasion—amidst

"the flow of soul"; it was a regular "Love" feast—all I. N. Love with themselves and others. Dr. Merryman is opposed to banquets and says they are all "*stuff.*"

---

TRANSACTIONS TEXAS STATE MEDICAL ASSOCIATION, '89.—This work is now in the bindery, and being rapidly finished. It is being bound in purple cloth and gilt, uniform with the editions of '85–'6, and will soon be in the hands of the members. It is a neat job, and in our next we will "review it."

By the bye, it would be time well spent if every member (officers especially) would read the Constitution, By-Laws and ordinances, and a better thing, if the officers would enforce them. There are requirements there of the existence of which even Presidents have seemed to be ignorant: for instance, Article X. of the by-laws prohibits a member or delegate from absenting himself from a meeting during a session, without special permission of the President, or from the session without the consent of the Association, the sense to be taken by vote. Members should remember this.

---

ON THE WAUGH-PATH!—The editor of the consolidated moloch of medical journals, i. e. the little Philadelphia fish which swallowed several larger fishes, and is now full to bursting, let his foot slip and said something about "clap trap" being "justifiable"; whereupon the *Drug Circular* goes for him red hot and says some pretty hard things about him. The editor of the C. M. of M. J. replies in invectives equally as choice, and lays the blame on some one else, saying the objectionable article "slipped in," and that it was intended for "irony,"—a "huge joke," etc., and winds up by asking his fellow-citizens to excuse him from replying any more to anything which the *Drug Circular* may say: he's *tired*; (ought to be,—very tired!)

*Moral*—Don't keep anybody or anything *slippery* about the office, and such jokes (?) will not slip in;—oughtn't even to wear slippers in the sanctum while the ironical (?) chap is about.

P. S.—Tell the i. c. hereafter to *label* his jokes.

A NEW DISCOVERY of vast importance has been made in London. A Mr. Wollhelm claims to have discovered a process by which sewer gas is instantaneously and thoroughly disinfected and deodorized. According to the Journal of the American Medical Association, he utilizes certain organic bases belonging to the group of ammonia compounds, in combination with lime. As the result of chemical reactions, a gaseous re-agent is evolved, to which he gives the name of "Amminol." It is a powerful disinfectant, and, according to the London Times, when introduced into sewage, rapidly extirpates all micro-organisms capable of causing putrefaction or disease. * * * In thirty minutes the liquid portion of the sewage can be discharged, deodorized and sterilized, with perfect safety.

The Journal adds: "According to reports made by Dr. Klein, F. R. S., the disinfection, as well as the deodorizing, is complete." "Dr. Klein suggests that the effects of the treatment on specific micro-organisms, such as bacillus anthracis, comma-bacillus of cholera, typhoid bacillus, etc., should be ascertained. We shall watch with interest for verification of Dr. Klein's experiments. If this new discovery can be utilized successfully, it may prove to be one of the most important achievements of the present age; and if its germicidal power can be used in the destruction of the microbes producing specific diseases, its value may be beyond any possible estimate. Its claims to our confidence will be absolute when this is fully verified."

It is now in order for Radam to rise up and get out an injunction for an infringement on the great original "Microbe Killer *de* Radam."

---

THE WEST TEXAS DISTRICT MEDICAL ASSOCIATION.—It will be remembered that this association was organized in 1876 and chartered. It is one of the oldest and best organizations in the State, and has done much and good scientific work; it has done much also to maintain the dignity of the profession of Texas. Until now it has held the meetings monthly,—but seeing the good work done by the Austin District, and the Central Texas, and the North Texas Medical Societies, "District" organizations, and being desirous of "keeping up their corner," this association recently resolved itself into a District Society, and in future will hold quarterly meetings,—the next to be at San Antonio on 30th October, prox.

We urge upon our readers resident in the region contiguous to San Antonio to attend this meeting, and to lend their aid in the laudable work undertaken. Only those who go to such meetings can realize the benefits derived; one learns something he did not know; gets an idea, perhaps, which may be of value to him and help him out of a tight place; makes the acquaintance of his confreres; forms new, and often lasting friendships; and returns to his labors after a day or so spent in social and scientific intercourse with the brethren, refreshed in mind and body; with a better opinion of himself and of mankind generally; with hope revived, and a determination to strive for higher and better things. It makes a new man of one, and—what is to be considered—it brings him into prominence in the profession.

Who are the best known physicians in Texas? Those who attend, reglarly, the meetings of the several associations, and contribute to their work. And, mark you, gentlemen,—we have made this prediction before—*the time will come* when not to be a member of some medical organization will be construed to mean that one *cannot be;* he will be an alien. The public are beginning to understand this thing, and to judge a physician by his standing with the profession. We know, for we are often interrogated by officers of insurance companies, for instance, as to the status of physicians in this State, with a view to appointing them as directors or examiners; and high estimate is put upon the fact that one is a member of the State or District Medical Society; it is equivalent to an endorsement in every respect,—whether justly in all cases, or not, it is a *prima facie* evidence of professional and moral standing.

We hope to chronicle the coming event, (October 30, meeting of the West Texas Medical Association,) with an account of a full meeting, and active and interesting work. God willing, we hope to be present. Dr. D. Berrey is Secretary. Write to him that you are coming.

